# The Fourth Branchial Complex Anomaly: A Rare Clinical Entity

**DOI:** 10.1155/2011/958652

**Published:** 2011-12-07

**Authors:** Alpen B. Patel, Michael L. Hinni

**Affiliations:** ^1^Department of Otolaryngology—Head and Neck Surgery, Mayo Clinic, 5779 East Mayo Boulevard, Phoenix, AZ 85054, USA; ^2^University of Arizona College of Medicine—Phoenix, Phoenix, AZ 85004, USA

## Abstract

Fourth branchial pouch anomalies are rare congenital disorders of the neck and are a consequence of abnormal development of the branchial apparatus during embryogenesis. Failure to appropriately recognize these anomalies may result in misdiagnosis, insufficient treatment, and continued recurrence. Here, we present an unique presentation of two cases, describe their diagnosis, clinical course, and management, and review the literature regarding these interesting anomalies.

## 1. Introduction

The human branchial apparatus, which develops in early gestation, is comprised of six paired mesodermal arches, separated by endodermal and ectodermal invaginations known as pouches and clefts, respectively [1]. Each arch is composed of a mesenchymal core, which is derived primarily from neural crest cells and will ultimately develop into the skeletal and interstitial structures of the head and neck along with associated vessels and nerves. Derivatives of the fourth pouch include the laryngeal cartilages, the laryngeal and pharyngeal constrictor muscles, the superior laryngeal nerve, the left thoracic aorta, the right proximal subclavian artery, the ultimobranchial body from which the calcitonin-secreting interfollicular cells of the thyroid arise, and the superior parathyroid glands [2–4]. 

Congenital lateral cervical cysts, fistulae, and sinuses are thought to arise from the branchial apparatus. Approximately 95% of congenital anomalies of the branchial apparatus involve the second branchial arch, pouch, or cleft, while the remaining mostly arise from the first and third arches [5]. Remnants of the fourth branchial arch are extremely rare with less than 100 cases reported in the literature [6] and account for 1–4% of all branchial anomalies [7]. These anomalies typically present as recurrent neck infections and/or abscesses or acute suppurative thyroiditis [5, 8]. 

The origin and course of the anomalies are determined by the mesodermal derivatives of adjacent arches; fistulae of the fourth branchial arch have an external opening in the neck on the anterior border of the lower sternocleidomastoid muscle and an internal opening in the pyriform sinus [1]. 

We present two rare cases of fourth branchial pouch abnormalities, the second of which represents a true, complete fistula, an anomaly rarely reported in the literature. The purpose of this manuscript is to present our experience with these cases, both of which demonstrate long-term followup. We have also emphasized the diagnosis, clinical course, and management of these interesting anomalies.

## 2. Case Report

**Case 1**

A 9-year-old white male presented with recurrent neck swellings, since the age of 2. During that time, he had a history of 27 procedures on his neck, ranging from explorations, drainages, and attempted excisions, with recurrence of signs and symptoms after every intervention. Upon presentation to our clinic, he complained of dysphagia and painful swelling of his neck.

Physical examination of his neck revealed swelling of the anterior left neck that was tender to palpation and was approximately 3 cm in diameter. Several computerized tomography (CT) scans had been performed in the previous years, all of which only revealed a diffuse anterior neck cystic structure, which was superficially located left of the midline.

**Figure 1:**
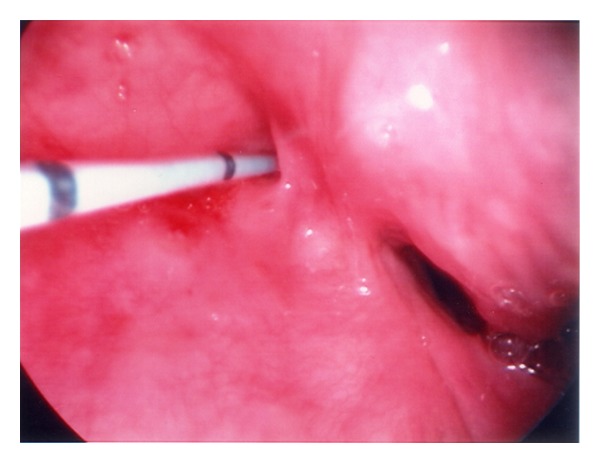
A sinus tract is seen originating from the left side of the pyriform sinus, while a cholangiocath was used to cannulate the tract prior to dissection.

In the operating room, direct laryngoscopy revealed a sinus tract originating from the left side of the pyriform sinus apex (Figure 1); a cholangiocath was used to cannulate the tract and surgical exploration of the neck revealed the tract to pass through the hypopharyngeal wall just posterior to the right laryngeal nerve entrance into the larynx and beneath the superior laryngeal nerve, consistent with a fourth branchial anomaly. The tract extended posteriorly and inferiorly, medial to the lesser cornu of the left thyroid ala. A portion of the posterior inferior thyroid cartilage was removed for exposure. All scar from previous surgery, the ipsilateral thyroid gland, and the tract were completely mobilized. The tract was severed at the entrance to the pyriform apex and oversewn. Postoperatively, the patient did well, and histopathology confirmed the presence of a sinus tract. The patient had an unremarkable postoperative course and has had no further recurrences during the past 8 years.

**Case 2**

A 42-year-old male presented with recurrent neck infections, left-sided neck pain, and a draining cutaneous pit to our clinic. The patient described a history of multiple procedures on his neck starting 1 year ago for what was thought to be a recurrent deep neck infection. He also recently became alarmed when blue fluid began emerging from his neck after drinking a blue-colored liquid, worrisome for a sinus tract. He was a previously healthy nonsmoker.

**Figure 2:**
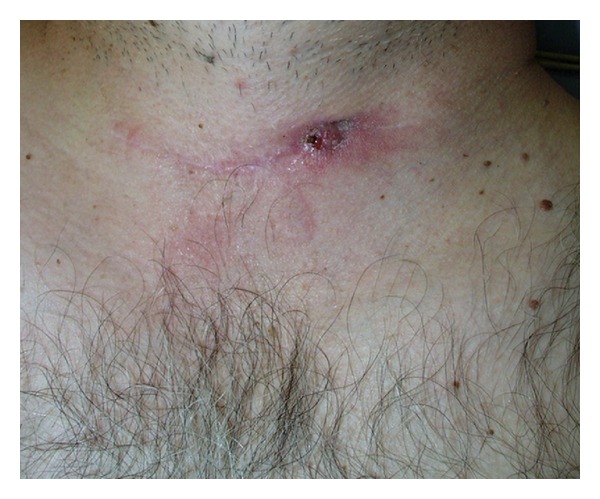
This fistula presented to our clinic as a draining neck wound on the anterior portion of the patient's left sternocleidomastoid muscle.

Flexible laryngoscopy revealed a small mucosal depression in the lateral posterior left pyriform sinus apex. He also had a draining neck wound on the anterior portion of his left sternocleidomastoid muscle (Figure 2). A barium swallow was performed, confirming the presence of a small sinus tract coming off the apex of the left pyriform sinus, consistent with a fourth branchial cleft anomaly.

**Figure 3:**
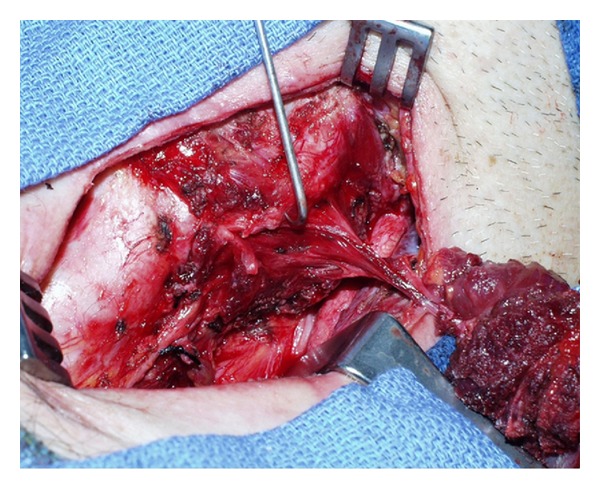
During dissection, the fistulous tract was seen exiting the pharynx inferior to the superior laryngeal nerve during exploration, while showing a complete connection with the neck skin.

In the operating room, a size-three foley catheter stylet was used to cannulate the tract. A curvilinear incision was made around the neck wound including the external fistula, the strap muscles were sectioned, and an oblique incision through the thyroid cartilage exposed the apex of the pyriform sinus, allowing the cannulated tract to become palpable. The fistulous tract was found to exit the pharynx inferior to the superior laryngeal nerve during exploration, consistent with an anomaly of the fourth branchial pouch (Figure 3). Histology confirmed a tract lined with columnar epithelium, further supporting our diagnosis of a remnant branchial sinus. The entire tract was excised along with surrounding thyroid tissue. The patient had an unremarkable postoperative course and has had no further recurrences during the past 10 years.

## 3. Discussion

Fourth branchial pouch anomalies are rare and usually present as lateral neck masses, abscesses, or acute suppurative thyroiditis [5]. These anomalies were first reported in 1972, and since then only sporadic cases have been reported, accounting for only 1–4% of all branchial apparatus anomalies [1]. Anomalies may be characterized as a fistula, sinus, or cyst: a fistula of branchial origin is composed of remnants of both pouch and cleft, with rupture of the interposed branchial plate; a sinus is a tract that is open to either gut or skin, but not both; a cyst is open to neither [9]. The third and fourth pouches are connected to the pharynx by pharyngobranchial duct. If this duct fails to degenerate in the seventh week of gestation, there is persistent communication with the pyriform fossa. Because of the similarity in their course, differentiating between anomalies of the major arches is often difficult on clinical grounds alone. The fistulous tract of a fourth branchial pouch originates within the pyriform sinus and descends to exit the pharynx inferior to the superior laryngeal nerve, cricothyroid muscle, and thyroid cartilage. The tract continues to descend lateral to the trachea and recurrent laryngeal nerve. On the left side, the tract curves forward under the arch of the aorta and then courses upward posterior to the internal carotid artery. On the right side, although rare, the tract circles forward underneath the subclavian artery before ascending. The tract proceeds superiorly, coursing over the hypoglossal nerve, to possible open externally in the neck at the lower anterior portion of the sternocleidomastoid muscle [3, 4, 10, 11]. There have only been a handful of cases in the literature claiming a true fourth pouch fistula; our second patient represents this finding with the tract being complete between the pyriform and the external neck skin. It is possible that both of these cases do not represent complete fistulae, but rather sinuses opened to the gut, which became infected and developed external drainage. This would explain the absence of significant chest extension.

A fistulous tract of a third branchial apparatus abnormality has a similar course to a fourth arch anomaly, but exits the pharynx superior to the superior laryngeal nerve [1, 10]. Particular to second branchial arch anomalies is their course which ascends superficial to the hypoglossal nerve and the stylopharyngeus muscle; moreover, they course superficial to the internal carotid artery, another distinguishing feature, and usually originate in the tonsillar fossa [12]. First branchial arch anomalies are considered to be duplications of the external auditory meatus and pinna with a sinus that runs parallel to the auditory meatus in type 1 anomalies or with a sinus that runs from an opening in the neck and ends blindly near the cartilaginous auditory meatus in a type 2 abnormality [13]. 

The common presenting symptoms of a fourth branchial pouch anomaly include recurring deep neck infections or abscesses, as well as soft fluctuant masses. Third and fourth branchial arch anomalies may also lead to acute suppurative thyroiditis, and for this reason, some authors recommend that in all cases of thyroiditis, the presence of a branchial arch anomaly should be investigated [8]. Diagnosis rests to demonstrate a sinus or fistula originating in the pyriform sinus, which should be performed. A barium esophagogram can depict these findings but should only be done after acute infection has resolved. CT and magnetic resonance imaging (MRI) are the modalities of choice for displaying both location and extent of pyriform sinus anomalies, as well as thyroid involvement [14, 15]. 

Our cases present an important issue in the treatment of fourth branchial pouch anomalies-prompt recognition. Both patients had undergone extensive medical treatment and surgical excisions to remove what were thought to be simple branchial cysts or thyroglossal duct cysts. The mainstay of treatment for fourth branchial anomalies is complete surgical excision because of the risk of infection and life-threatening abscesses [6, 8]. The use of endoscopic cauterization limited to the sinus tract origin as a less-invasive procedure has been noted [16]. Recently, use of sclerotherapy with OK-432 has been expanded to treat branchial cleft cysts [17]. Because of the high incidence of secondary infection of these anomalies, early excision is recommended. The surgical approach should begin with exposure of the thyroid ala and carotid sheath to begin in an area free of postinflammatory fibrosis [6]. As utilized by our team, a thorough exam of the airway is critical, and cannulation of the tract under direct visualization with a small catheter is very helpful in aiding a complete and safe dissection [11]. Due to the intimacy of tracheal structures and fibrosis, it is often ideal to remove a portion of the thyroid gland as well [3, 4].

## 4. Conclusion

Fourth branchial arch anomalies are rare and fascinating aberrations of fetal development that may present in many different ways. Combining a proper preoperative evaluation with careful surgical planning may result in the proficient eradication of these lesions, offering the patient relief from this source of recurrent infection. Definitive management is achieved by complete surgical excision of the anomaly.

##  Conflict of Interest

The authors have no conflict of interest to disclose.
